# Classification of subtypes including LCNEC in lung cancer biopsy slides using convolutional neural network from scratch

**DOI:** 10.1038/s41598-022-05709-7

**Published:** 2022-02-03

**Authors:** Jung Wook Yang, Dae Hyun Song, Hyo Jung An, Sat Byul Seo

**Affiliations:** 1 Department of Pathology, Gyeongsang National University Hospital, Jinju, Republic of Korea; 2 Department of Pathology, Gyeongsang National University, College of Medicine, Jinju, Republic of Korea; 3 Gyeongsang Institute of Health Science, Jinju, Republic of Korea; 4 Department of Pathology, Changwon Gyeongsang National University Hospital, Changwon, Republic of Korea; 5 Department of Mathematics Education, School of Education, Kyungnam University, 7 Kyugnamdaehak-ro, Masanhappo-gu, Changwon, Gyeongsangnam-do 51767 Republic of Korea

**Keywords:** Lung cancer, Machine learning

## Abstract

Identifying the lung carcinoma subtype in small biopsy specimens is an important part of determining a suitable treatment plan but is often challenging without the help of special and/or immunohistochemical stains. Pathology image analysis that tackles this issue would be helpful for diagnoses and subtyping of lung carcinoma. In this study, we developed AI models to classify multinomial patterns of lung carcinoma; ADC, LCNEC, SCC, SCLC, and non-neoplastic lung tissue based on convolutional neural networks (CNN or ConvNet). Four CNNs that were pre-trained using transfer learning and one CNN built from scratch were used to classify patch images from pathology whole-slide images (WSIs). We first evaluated the diagnostic performance of each model in the test sets. The Xception model and the CNN built from scratch both achieved the highest performance with a macro average AUC of 0.90. The CNN built from scratch model obtained a macro average AUC of 0.97 on the dataset of four classes excluding LCNEC, and 0.95 on the dataset of three subtypes of lung carcinomas; NSCLC, SCLC, and non-tumor, respectively. Of particular note is that the relatively simple CNN built from scratch may be an approach for pathological image analysis.

## Introduction

Lung cancer is most common cause of cancer death (18.0%) while it was the second most commonly diagnosed cancer (2,206,771; 11.4%) worldwide in 2020 behind female breast cancer (2,261,419; 11.7%)^1^. The operability of lung cancer is determined by the cancer stage. At the time of diagnosis, approximately 70% of lung cancer patients have advanced stages and are inoperable. In many cases, a final histological diagnosis is made from a small biopsy tissue. After a small biopsy, lung carcinomas are classified as adenocarcinoma (ADC), squamous cell carcinoma (SCC), small cell lung cancer (SCLC), large cell neuroendocrine carcinoma (LCNEC), or “non-small cell carcinoma (NSCC), not otherwise specified (NOS)”, etc. by additional special and/or immunohistochemical stains, if necessary. The classification “NSCC, NOS” is the diagnostic term used when a lung carcinoma cannot be classified into a specific type from a small biopsy specimen despite the use of the additional stains or when no stains are available^2^. Subtyping for lung carcinoma in small biopsy specimens is often challenging without these special and/or immunohistochemical stains.

Histological subtyping of lung carcinoma is a vital part of determining a suitable treatment plan. In cases of small cell lung cancer, chemotherapy is usually used alone without any surgical treatment because of the advanced cancer stage at the time of diagnosis; it is hard to meet surgical specimens of small cell lung cancer in practice. In the patients with NSCC, surgery is determined by the cancer stage, and the chemotherapy regimen depends on the histological subtype^3,4^.

With the emergence of digital pathology, there have been studies that attempt to analyze digital slide images of lung cancers using deep learning^5–12^. Deep learning-based pathology image analysis that can accurately diagnose and subtype lung carcinoma would be useful in daily practice as an auxiliary means. There are several deep learning studies that have proposed technologies that are capable of distinguishing non-neoplastic lung tissue and lung carcinoma subtype including adenocarcinoma, squamous cell carcinoma, and small cell lung cancer, which are frequently seen in lung biopsy^8,10,12^, however, LCNEC was not included in the studies.

Previous studies mostly have used single or mixed open-sourced convolutional neural network (CNN) models such as AlexNet^13^, GoogLeNet^14^, ResNet^15^, VGGNet^16^, which were benchmarked on ImageNet dataset. A simple CNN model dedicated to a specific task, such as pathological image analysis, could produce more efficient results. In this study, we propose a CNN model built from scratch along with pre-trained CNN models for classification of subtypes including LCNEC in lung cancer biopsy slides as an efficient method for pathology image analysis.

## Results

### Overview of AI models for classification of lung cancer subtypes

**Figure 1:**
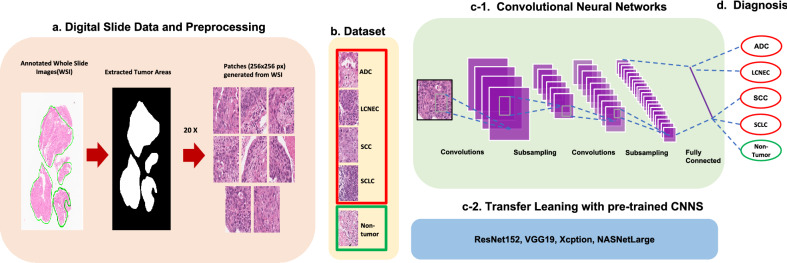
Workflow of AI model for multinomial pattern classification in lung cancer biopsies. (**a**) Digital slide data and preprocessing. Cancer regions on the whole slide images (WSIs) were annotated by pathologists, then the tumor areas were extracted and used to generate non-overlapping patches 256 × 256 pixels in size at a magnification of 20 × . (**b**) Dataset. The dataset contains a total of 5 classes with 21 of ADC, 14 of LCNEC, 22 of SCC, and 21 of SCLC WSIs, as well as 127 of non-tumor WSIs. 10,049 patches were generated from the original 205 WSIs containing the four lung cancer subtypes and negative cases. (**c-1**) Convolutional Neural Network. The model takes a tensor with dimensions of (244, 244, 3) as input, the CNN consists of four convolution blocks with a max pool layer in each. The 1st and 2nd hidden layer have 16 and 32 filters, respectively, with a kernel size of (2, 2) and use a rectified linear unit (ReLU) as their activation functions. The 3rd and 4th hidden layer have 64 filters with a kernel size of (2, 2) and also use rectified linear units (ReLU). The CNN also contains a fully connected dense layer with 5 units that uses softmax as its activation function. When compiling the model, Nadam and categorical cross entropy were chosen as the optimizer and loss function, respectively. (**c-2**) Transfer Learning with pre-trained CNNs. Four pre-trained convolutional neural networks based on ResNet152^15^, VGG19^16^, Xception^17^, and NASNETLarge^18^ were evaluated in this study. After the pre-trained models were chosen, we repurposed their already learned knowledge and carried out fine-tuning for our task. (**d**) Diagnosis. The AI models diagnosed the input images from the test sets as either one of four types of lung cancer or as a negative case.

**Figure 2:**
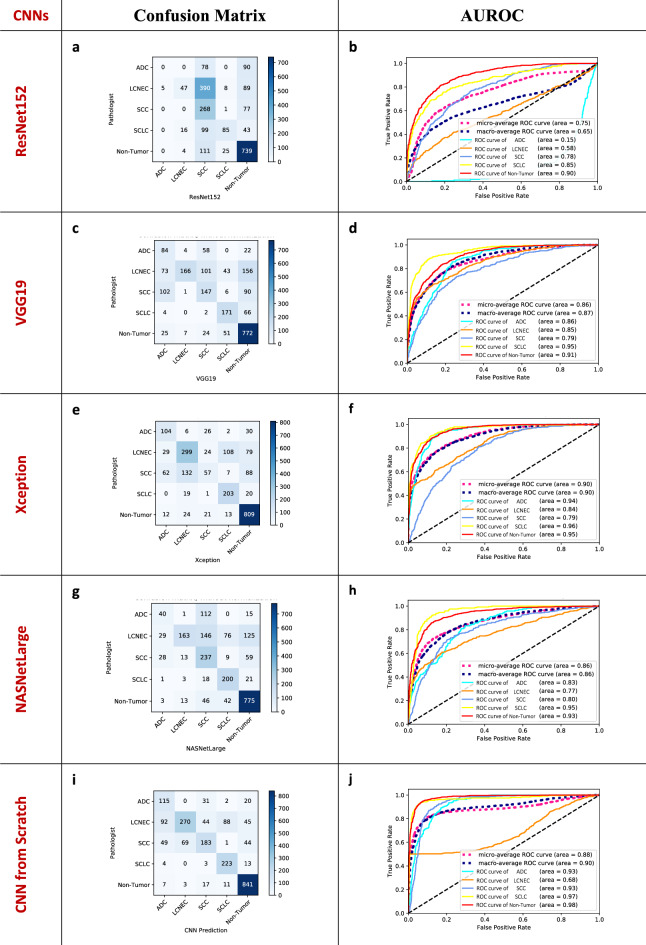
Confusion matrices and areas under the curve (AUC) for the receiver operating characteristic curves (ROC) achieved by the AI models for the multi-classification**.** To evaluate the performance of each multi-classification model, normalized confusion matrices (**a**,**c**,**e**) and areas under the curve (AUC) for the receiver operating characteristic curve (ROC) (**b**,**d**,**f**) were calculated for each model on the test set [ResNet152 (**a**,**b**), VGG19 (**c**,**d**), Xception (**e**,**f**), NASNetLarge (**g**,**h**), CNN from scratch (**i**,**j**)]. Each row of each matrix represents the number of patches in a predicted class by the corresponding AI model, while each column represents the actual instances in each class according to pathologists.

We developed and evaluated AI models with a convolutional neural network structure for multi-classification of lung cancer subtypes (Table 2, Fig. 1c-1) either built from scratch on the Keras Sequential API (https://keras.io/) or based on four well-known, pre-trained CNNs using transfer learning (Fig. 1c-2). Pathology slides of lung or bronchus biopsies from 203 patients and surgical specimens from 2 patients were collected at the Gyeongsang National University Hospital. Cancer regions on the 205 WSIs were annotated by pathologists, and the tumor areas in these images were extracted and used to generate non overlapping patches 256 × 256 pixels in size at a magnification of 20 × using DeepPATH^7^, as shown in Fig. 1a. Figure 1b shows the dataset containing a total of 5 classes; 21 of ADC, 14 of LCNEC, 22 of SCC, 21 of SCLC whole slide images (WSIs), and 127 of non-tumor WSIs. 10,049 sample patch images were generated from 205 WSIs to represent the four lung cancer subtypes as well as the negative case. Out of the total of 9849 patches, 7089 patches were used to construct a training set, 785 patches were selected for a validation set, and 2175 patches were assigned to the test set as shown in Table 1. Test set consisted of 168 patches from 3 of ADC slides, 539 patches from 2 of LCNEC slides, 346 patches from 3 of SCC slides, 243 patches from 3 of SCLC slides, and 879 patches from 11 of non-tumor slides as shown in Table 1. The architecture of the CNN built from scratch is described in Table 2. Four pre-trained convolutional neural networks, ResNet152^15^, VGG19^16^, Xception^17^, and NASNETLarge^18^, were also used in this study. To compile the model for each CNN, nadam and categorical cross entropy were chosen as the respective optimizer and loss function. Each AI model diagnosed the images and output either one of the four types of lung cancer or the negative case using the two test sets, as shown in Fig. 1d.

**Table 1:** Summary of number of whole slide images (WSIs) and patches in the dataset: training, validation, and test set among four lung cancer subtypes and a negative case.

Subtypes	Total (n)		Training set		Validation set	Test set	
	WSIs	Patches	WSIs	Patches	Patches	WSIs	Patches
**Tumor**							
ADC	21	1573	18	1265	140	3	168
LCNEC	14	1386	12	763	84	2*	539
SCC	22	1397	19	946	105	3	346
SCLC	21	1610	18	1231	136	3	243
Non-tumor	127	4083	116	2884	320	11	879
Total	205	10,049	183	7089	785	22	2175

*ADC* adenocarcinoma, *LCNEC* large cell neuroendocrine carcinoma, *SCC* squamous cell carcinoma, *SCLC* small cell lung cancer.

*Surgical specimen.

**Table 2:** Summary of 2D CNN model architecture.

Layer (options)	Output shape	Number of parameters
Input	(None, 224, 224, 3)	–
Conv2D (filters = 16, kernel_size = (2,2), activation = ‘relu’)	(None, 223, 223, 16)	208
MaxPooling2D	(None, 111, 111, 16)	0
Conv2D (filters = 32, kernel_size = (2,2), activation = ‘relu’)	(None, 110, 110, 32)	2080
MaxPooling2D	(None, 55, 55, 32)	0
Conv2D (filters = 64, kernel_size = (2,2), activation = ‘relu’)	(None, 54, 54, 64)	8256
MaxPooling2D	(None, 27, 27, 64)	0
Conv2D (filters = 64, kernel_size = (2,2), activation = ‘relu’)	(None, 26, 26, 64)	16,448
MaxPooling2D	(None, 13, 13, 64)	0
Global Average Pooling2D	(None, 64)	0
Dense (unit = 5, activation = ‘softmax’)	(None, 5)	325

### Diagnostic performances of AI models for multi-classification

To evaluate the diagnostic performance of each multi-classification AI model, confusion matrices and the area under the curve (AUC) of the receiver operating characteristic curve (ROC) for each model over the test sets were calculated as shown in Fig. 2. For the confusion matrices, each row of one of the matrices represents the number of patches in each predicted class according to the AI models, while each column represents the actual number of instances in each class according to the pathologists. Table 3 displays the classification results of CNN models over the test set; precision, recall, f1-score, and accuracy. The ResNet152 model achieved AUC of 0.65 for the macro-average ROC curve, its AUCs for classifying subtypes of lung cancer ranged from 0.15 to 0.90 on the test set, as shown in Fig. 2b. It produced the weighted average of precision, recall, and f1-score of 0.5384, 0.5237, and 0.4685 as shown in Table 3. The VGG19 model achieved AUC of 0.87 for the macro-average ROC curve, and its AUCs for classifying subtypes of lung cancer ranged from 0.79 to 0.95 on the test set, as shown in Fig. 2d. The weighted average of precision, recall, and f1-score of the VGG19 were 0.6767, 0.6161, and 0.6009, respectively. Figure 2e,f show the confusion matrix and AUROC of the Xception model. The Xception model achieved AUC of 0.90 for the macro-average ROC curve, which is the highest among the four pre-trained CNN models, and its AUCs for classifying each cancer subtype ranged from 0.79 to 0.95. The weighted average of precision, recall, and f1-score of the Xception were 0.6502, 0.6708, and 0.6485, respectively. The NASNetLarge model achieved the AUC of 0.86 for the macro-average ROC curve, and its AUCs for classifying subtypes of lung cancer ranged from 0.77 to 0.93 on the test set, as shown in Fig. 2d. The weighted average of precision, recall, and f1-score of the NASNetLarge were 0.6904, 0.6506, and 0.6293, respectively. Figure 2i,j showed diagnostic performance of the CNN built from scratch. It produced an AUC of 0.90 for the macro-average ROC curve, its AUCs for classifying subtypes of lung cancer ranged from 0.68 to 0.98. The weighted average of precision, recall, and f1-score of the scratch CNN were 0.7632, 0.7503, and 0.7428, respectively. The model Xception and ResNet152 models had the highest and the lowest performance among the pre-trained CNNs. The CNN built from scratch showed similar performance to the model with Xception as the results of AUC, but showed better performance than the Xception model considering overall diagnostic performance (i.e. precision, recall f1-score, and accuracy). Table 4 displays the detailed classification results (i.e. the precision, recall, f1 score, and accuracy) of the CNN models for each class.

**Table 3:** Performance of the CNN models.

Models	Classification	Precision	Recall	f1-score	Accuracy	Support
ResNet152	ADC	0.0000	0.0000	0.0000	0.5237	168
	LCNEC	0.7015	0.0872	0.1515		539
	SCC	0.2833	0.7746	0.4149		346
	SCLC	0.7143	0.3498	0.4696		243
	Non-tumor	0.7119	0.8407	0.7710		879
	Macro avg	0.4822	0.4105	0.3621		2175
	Weighted avg	0.5384	0.5237	0.4685		2175
VGG19	ADC	0.2917	0.5000	0.3684	0.6161	168
	LCNEC	0.9326	0.3080	0.4630		539
	SCC	0.4428	0.4249	0.4336		346
	SCLC	0.6310	0.7037	0.6554		243
	Non-tumor	0.6980	0.8730	0.7778		879
	Macro avg	0.5992	0.5630	0.5417		2175
	Weighted avg	0.6767	0.6161	0.6009		2175
Xception	ADC	0.5024	0.6190	0.5547	0.6708	168
	LCNEC	0.6229	0.5447	0.5868		539
	SCC	0.4419	0.1647	0.2400		346
	SCLC	0.6096	0.8354	0.7049		243
	Non-tumor	0.7885	0.9204	0.8493		879
	Macro avg	0.5931	0.6189	0.5871		2175
	Weighted avg	0.6502	0.6708	0.6485		2175
NASNetLarge	ADC	0.3960	0.2381	0.2974	0.6506	168
	LCNEC	0.8446	0.3204	0.4454		539
	SCC	0.4240	0.6850	0.5238		346
	SCLC	0.6116	0.8230	0.7018		243
	Non-tumor	0.7789	0.8817	0.8271		879
	Macro avg	0.6110	0.5860	0.5591		2175
	Weighted avg	0.6904	0.6506	0.6293		2175
CNN from scratch	ADC	0.4307	0.6845	0.5287	0.7503	168
	LCNEC	0.7895	0.5009	0.6129		539
	SCC	0.6583	0.5289	0.5865		346
	SCLC	0.6862	0.9177	0.7852		243
	Non-tumor	0.8733	0.9568	0.9131		879
	Macro avg	0.6876	0.7178	0.6853		2175
	Weighted avg	0.7632	0.7503	0.7428		2175

*ADC* adenocarcinoma, *LCNEC* large cell neuroendocrine carcinoma, *SCC* squamous cell carcinoma, *SCLC* small cell lung cancer, *Precision* the fraction of relevant instances among the retrieved instances, *Recall* the fraction of relevant instances that were retrieved, *f1-score* a measure of test set’s accuracy and the harmonic mean of the precision and recall, *Support* number of test set for each label and total, *weighted average* averaging the support-weighted mean per label, *macro average* the arithmetic mean of individual classes’ precision, recall, and f1-scores.

**Table 4:** Performance of the CNN from scratch model for test sets.

Dataset	Classification	Precision	Recall	f1-score	Accuracy	Support
Dataset	ADC	0.4307	0.6845	0.5287	0.7503	168
	LCNEC	0.7895	0.5009	0.6129		539
	SCC	0.6583	0.5289	0.5865		346
	SCLC	0.6862	0.9177	0.7852		243
	Non-tumor	0.8733	0.9568	0.9131		879
	Macro avg	0.6876	0.7178	0.6853		2175
	Weighted avg	0.7632	0.7503	0.7428		2175
Dataset′	ADC	0.5442	0.7321	0.6244	0.8674	168
	SCC	0.8405	0.7312	0.7821		346
	SCLC	0.9187	0.9300	0.9243		243
	Non-tumor	0.9467	0.9295	0.9380		879
	Macro avg	0.8125	0.8307	0.8172		1636
	Weighted avg	0.8788	0.8674	0.8708		1636
Dataset″	NSCLC	0.9379	0.8604	0.8975	0.8920	1053
	SCLC	0.6257	0.9424	0.7521		243
	Non-tumor	0.9549	0.9158	0.9350		879
	Macro avg	0.8395	0.9062	0.8615		2175
	Weighted avg	0.9099	0.8920	0.8964		2175

*ADC* adenocarcinoma, *LCNEC* large cell neuroendocrine carcinoma, *SCC* squamous cell carcinoma, *SCLC* small cell lung cancer, *Dataset*′ the dataset excluding the class LCNEC, *Dataset*″ the dataset consisting of NSCLC (ADC, LCNEC, SCC), SCLC, and non-tumor, *Precision* the fraction of relevant instances among the retrieved instances, *Recall* the fraction of relevant instances that were retrieved, *f1-score* a measure of test set’s accuracy and the harmonic mean of the precision and recall, *Support* number of test set for each label and total, *weighted average* averaging the support-weighted mean per label, *macro average* the arithmetic mean of individual classes’ precision, recall, and f1-scores.

### Predictive analysis of CNN from scratch for discriminating patterns

**Figure 3:**
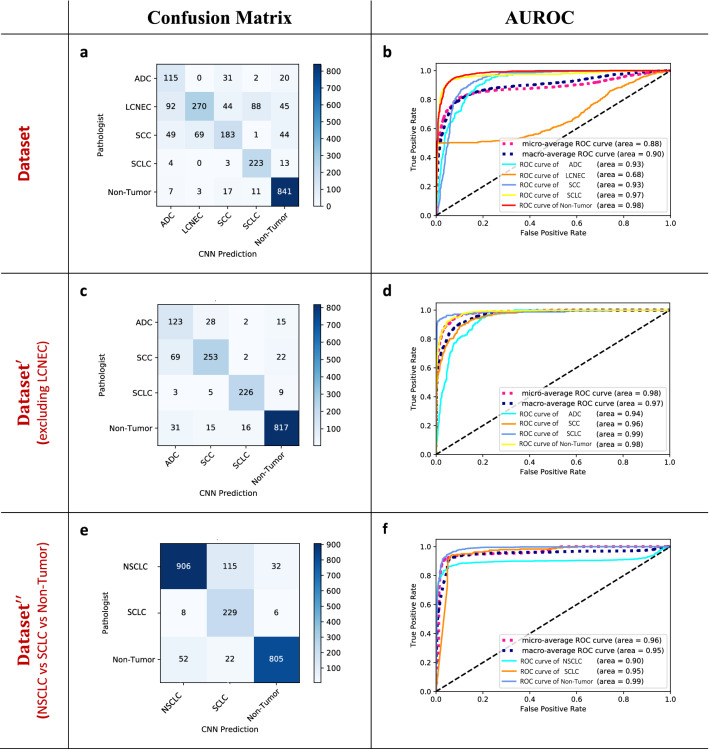
Confusion matrices and areas under the curve (AUC) for the receiver operating characteristic curves (ROC) achieved by the CNN from scratch on the three data sets. The diagnostic performance of the CNN model built from scratch in multinomial classification of lung cancer subtypes was evaluated on the test sets. Dataset consisted of five classes; ADC, LCNEC, SCC, SCLC, and non-tumor. Dataset′ consisted of four classes excluding LCNEC from the dataset. Dataset″ composed three classes with NSCLC (combining ADC, LCNEC, and SCC), SCLC, and non-tumor. The area under the curve (AUC) of receiver operating characteristic curve (ROC) achieved by the scratch CNN model on each dataset are shown [Dataset (**a**,**b**), Dataset′ (**c**,**d**), Dataset″ (**e**,**f**)].

We evaluated the diagnostic performance of the AI models for multinomial classification of lung cancer subtypes on the test sets. Dataset′ consisted of four classes excluding LCNEC from the original dataset, and dataset″ composed three classes with NSCLC (combining ADC, LCNEC, and SCC), SCLC, and non-tumor. The confusion matrices and the area under the curve (AUC) of the receiver operating characteristic curve (ROC)s were evaluated for the CNN model built from scratch over the test sets as shown in Fig. 3. Table 4 displays the classification results of the CNN from scratch over the test sets. The built from scratch CNN model produced the AUC of 0.90 for the macro-average ROC curve, its AUCs for classifying subtypes of lung cancer ranged from 0.68 to 0.98 in the original test set as shown in Fig. 3a,b. On the other hand, the macro-average AUC of the model raised up to 0.98 on the test set from the dataset′, which excluded the class of LCNEC (see in Fig. 3c,d). Table 3 showed that the weighted average of precision, recall, and f1-score of the scratch CNN raised from 0.7632 to 8788, from 0.7503 to 8674, and from 0.7428 to 8708, respectively as the dataset was excluded LCNEC. The prediction analysis of the scratch CNN for the test set from the dataset reconstructed into new classes (NSCLC, SCLC, and non-tumor; dataset″) were evaluated as shown in Table 4 and Fig. 3e,f. The scratch CNN model produced the AUC of 0.96 for the macro-average ROC curve, its AUCs for classifying subtypes of lung cancer ranged from 0.90 to 0.99 as shown in Fig. 3e,f. The weighted average of precision, recall, and f1-score of the scratch CNN model for the dataset″) were 0.9099, 0.8920, and 0.8964, respectively as Table 3.

## Discussion

In this work, we generated various deep learning-based AI models for classification of subtypes in lung cancer biopsy slides. The AI models for the classification of subtype lung carcinoma proceeded with the flow of digital WSI data preparation, convolutional neural networks, loss functions, and diagnostic performance evaluation. Previous research involving lung cancer image analysis with deep learning has focused on differentiating between non-cancer tissue and lung cancer^5,6^ to distinguish adenocarcinoma, squamous cell carcinoma^7^, or looked to identify small cell carcinoma in addition to the cancers previously mentioned^8,10^. Gonzalez et al. attempted to distinguish LCNEC from small cell carcinoma using deep learning, however, this is based on cytology images as the input information^9^. We could not find any studies that have attempted to classify the major carcinoma subtypes including LCNEC, using lung biopsy images as the input.

In this study, we took two approaches to developing AI models that distinguish multinomial patterns of lung carcinoma: transfer learning with pre-trained CNNs and a built from scratch CNN. First, we used transfer learning with pre-trained convolutional neural networks based on ResNet152^15^, VGG19^16^, Xception^17^, and NASNETLarge^18^. Among those pre-trained CNN models, Xception achieved the highest overall performance and ResNet152 produced the lowest performance. These results demonstrate that transfer learning with pre-trained CNNs on ImageNet^19^ is relatively easy to access and ensures obtaining a certain level of verified accuracy. However, these results also imply, even among the pre-trained CNN models, the performances of each model may vary in pathology image analysis, as shown in Table 3. The model with ResNet152 achieved the lowest performance despite being known as proven performance. ResNet152 showed significantly lower performance, especially for ADC and LCNEC, than the results of other models. In addition, an interesting result is that ResNet15 misclassified the SCLC cases as well as most cases of ADC and LCNEC, while the other models easily diagnosed the class of SCLC. ResNet152 may require more fine-tuning to analyze for pathology images. The previous studies have tended to use pre-trained CNNs on a large benchmark dataset, for example, ImageNet^19^. However, our dataset consisted only of the scanned hematoxylin–eosin stained pathology slides, which is away from the ImageNet^19^. Our results imply that using a pre-trained CNN with proven performance is one method, but it does not necessarily guarantee high performance. In other words, it may be crucial to choose an appropriate model for the specific task, such as analyzing pathological images.

The second approach was to customize a new convolutional neural network model. The built from scratch CNN model performed similar or better than the transfer learning using pre-trained CNNs on a large benchmark dataset. The CNN built from scratch showed similar performance to the model with Xception as the results of AUC, but showed better performance than the Xception model considering overall diagnostic performance (i.e. precision, recall f1-score, and accuracy). These results imply that the built from scratch CNN model fitted to the specific task at hand with a certain amount of data, such as pathological diagnosis of lung cancer subtypes, could be expected to produce better performances rather than that of transfer learning using the pre-trained CNNs.

For each class, Xception, NASNetLarge, and CNN from scratch were relatively good at distinguishing the classes of SCLC and non-tumor tissue, but not at distinguishing NSCLC subtypes (ADC, LCNEC, SCC; see in Table 3). Although it did not show that the performance of the scratch CNN was superior for ADC, LCNEC, and SCC to others, overall, those were similar or better than the performance of the other models.

The AUC for LCNEC on the CNN from scratch was exceptionally low (AUC = 0.68) compared to others (see in Fig. 2j). Although the reason for the low AUC for LCNEC on our model is not clearly known, the use of surgical specimens in the test set may be one possible reason. Further studies are needed for degree of performance on deep-learning models trained with lung cancer biopsy slides depending on sample subtypes.

In additional analyses, the CNN from scratch showed better performance for distinguishing 4 classes (excluding LCNEC) and 3 classes (NSCLC, SCLC, and non-tumor) as shown in Table 4. For classification excluding LCNEC, the CNN from scratch showed similar AUCs (0.94 for ADC, 0.96 for SCC, and 0.99 for SCLC) with Kanavati’s study^10^ (0.814–0.987 for ADC, 0.959–0.989 for SCC, and 0.994–0.999 for SCLC). The performance change when LCNEC is not classified can be interpreted as follows. One possible reason is that classification of LCNEC makes prediction of other cancer subtypes difficult. Actually, distinguishing among poorly differentiated ADC, poorly differentiated SCC, and LCNEC is challenging without immunohistochemistry, and LCNEC may also be difficult to distinguish from SCLC in biopsy in some cases. Travis et al. reported that unanimous agreements in LCNEC and SCLC were 40% and 70% of cases, respectively, and the most common disagreement occurred between LCNEC and SCLC in surgically resected pulmonary neuroendocrine tumors reviewed independently by five pulmonary pathologists^20^.

Another possibility is increasing the number of classes may cause the prediction to be more challenging in general.

There are some limitations to our study. First, our data set may be a relatively small number of samples to train and test the models for classifying into five classes. The number of samples in each class might be small, especially in the class of LCNEC. In addition, LCNEC cases in the test set were surgical specimens. However, even with relatively small data, a similar rate of performance results was obtained compared to previous studies with large amounts of data. This suggests that, in the development of AI models, finding a quantitative criterion of data for training and performance testing should be conducted in future research. Secondly, our study was conducted at patch level compared to the actual pathological diagnosis performed at slide level. The diagnoses of biopsy are more difficult than that of surgical specimens for pathologists. Therefore, it appears that NSCLC classification performance is lower than expected, as it may be more difficult to differentiate cancer subtypes at the patch level than at the slide level. Diagnosis of AI models at slide-level is generally performed first at the patch level. A class is determined first at the patch level, and then diagnosed at slide level by the proportion of the certain class patches on the slide. Thus, the performance of AI models at the slide level should be similar or better than that of at the patch level. In previous studies^10^ have shown that the prediction rate of the AI model at the patch level was similar to or lower than the prediction rate at the slide level. This study was conducted to evaluate the optimal diagnostic performance at the patch level. The third limitation of our study is that our dataset is “controlled’ data, not “real-world” data. The dataset consisted of lung or bronchus biopsy slides from Gyeongsang National University Hospital in 2012. Due to the relatively small number of LCNEC biopsy cases (n = 2) in 2012, we added more LCNEC biopsy cases from 2013 to 2018 to ensure appropriate analysis for LCNEC and to balance among the diagnoses. Our dataset does not represent real-world distribution of diagnoses, so this may produce lower performance on real-world dataset. However, small-scale research is impossible if only real-world data is used when AI pathology studies for cancers with a low incidence rate such as pulmonary LCNEC. Lastly, our models were built using an intra-hospital dataset. In other studies, public data such as the TCGA dataset was used to classify lung cancer versus normal tissue or the multinomial classification of the various cancer subtypes^4,5^, however, at the time of writing, there are no public databases that include LCNEC and small cell carcinoma slides available.

In conclusion, we compared two types of deep learning-AI models for classification of subtypes including LCNEC in lung cancer biopsy slides. The first type of model was a CNN pre-trained using transfer learning, and our experiments showed that this model was able to consistently classify the various classes of lung cancer type at a certain level of verified accuracy using actual pathological data as the input. The performance achieved in the experiments conducted demonstrates that a CNN model has the potential to be the basis for developing diagnostic workflow systems of the diagnosis and subtyping of lung cancers. However, the pre-trained CNNs on ImageNet^19^ are generally complicated and it requires a time-consuming process to run. In addition, expensive equipment and lots of electrical energy are indispensable for experiments. This seems to be challenging for AI models to apply and practical use to all hospitals immediately. Nevertheless, studies on AI in pathology have been actively conducted, and it will be expected to continue and produce a lot of progress. The information gathered in this study suggests that one approach to pathology image analysis is to use a relatively simple CNN built from scratch model fitted to the specific task, like the model demonstrated in this paper.

## Materials and methods

### Patients

We collected hematoxylin–eosin stained pathology slides from the pathology reports of 171 patients that underwent lung or bronchus biopsies at Gyeongsang National University Hospital, Jinju, Korea, in 2012, and the pathology slides of 12 patients diagnosed with large cell neuroendocrine carcinoma in their biopsy at Gyeongsang National University Hospital from 2012 to 2018. Out of the patients, the pathology slides were taken from 18, 19, and 18 of patients were respectively diagnosed with adenocarcinoma, squamous cell carcinoma, and small cell carcinoma, while other slides (n = 116) all came from non-tumor cases. Those slides were constructed as the training and validation in the dataset as shown in Table 1. The test set was composed of additional cases from 2013 and 2015 (3 of ADC, 2 of LCNEC, 3 of SCC, 3 of SCLC, and 11 of non-tumor slides). Among these slides, the two cases of LCNEC (one was from 2013, the other was from 2015) were surgical specimens. The biopsy was not done for the two cases, and those were entirely independent cases from the slides in the training and validation set. Each diagnosis was histopathologically confirmed by two experienced pathologists. This study was approved by the Institutional Review Board of Gyeongsang National University Hospital with a waiver for informed consent (2021-04-016), and all methods were performed in accordance with the relevant guidelines and regulations.

### Data preprocessing

A total of 205 WSIs were acquired from 205 pathology slides with an Aperio AT2 slide scanner (Leica Biosystems Division of Leica Microsystems Inc., IL, USA) and 400 ×. Two experienced pathologists annotated the cancer regions on the WSIs with Aperio ImageScope v12.4.3 (Leica Biosystems Division of Leica Microsystems Inc., IL, USA). Tumor areas were extracted from the annotated whole slide images (WSIs), the extracted areas were used to generate non-overlapping patches 256 × 256 pixels in size at a magnification of 20 × using DeepPATH based on the OpenSlide library in Python (Fig. 1a)^7^. The patches were removed if the percentage of background in the patch was above 25% according to the DeepPATH^7^ program. In this process, 10,049 patches were generated from the original 205 WSIs containing either one of the four lung cancer subtypes or a negative case, as shown in Table 1.

### Patch dataset

The dataset consisted of slides from patients in one of 5 classes with 21 of ADC, 14 of LCNEC, 22 of SCC, and 21 of SCLC whole slide images (WSIs) of lung cancer subtypes as well as 127 of non-tumor WSIs. The training and validation set contained 3 of ADC, 2 of LCNEC, 3 of SCC, 3 of SCLC, and 11 of non-tumor slides. These WSIs generated 7089 of patches and 785 of patches in the training and validation set, respectively. The test set was generated by the additional slides; 3 of ADC, 2 of LCNEC, 3 of SCC, 3 of SCLC, and 11 of non-tumor slides independent from the training and validation set. The test set consisted of 2175 patches in five classes. The detailed information of the dataset is seen in Table 1. To compare the performance of AI models, we reconstructed the dataset into dataset′ and dataset″. Dataset′ consisted of only four classes excluding LCNEC from the original dataset in Table 1. Dataset″ is the dataset consisting of three classes; NSCLC, SCLC, and non-tumor. The class of NSCLC was generated by combining ADC, LCNEC and SCC patches from the dataset in Table 1.

### Convolutional neural networks

A deep neural network (DNN) is a supervised classifier that contains multiple layers between input and output layers^21^. A convolutional neural network (CNN, or ConvNet) is a specialized kind of a DNN, CNNs are known to perform particularly when analyzing images^19–22^. We constructed a convolutional neural network model for the multinomial classification of lung cancer biopsies with the possible outputs being the four lung cancer types or the negative case. Our CNN was built on the Keras Sequential API (https://keras.io/), written in Python and running on TensorFlow (https://www.tensorflow.org/)^23^. CNN models take tensors of a certain shape as input, for image analysis CNNs the shape of these tensors are dictated by the height of input images, width, and color channels. Our model takes inputs with dimensions of 244 × 244 × 3 and consists of four convolution blocks with a max pool layer in each. The 1st and 2nd hidden layers of the model have 16 and 32 filters, respectively, with a kernel size of (2, 2) and use a rectified linear unit (ReLU) as their activation functions. The 3rd and 4th hidden layers have 64 filters with a kernel size of (2, 2) and use a rectified linear unit (ReLU) as their activation functions, as shown in Table 2. The fully connected dense layer of the model has 5 units and uses a softmax activation function. Batch size of 200 and 100 epochs were determined as the optimum values for the model when considering both time and computational costs. When compiling the model, Nadam was chosen as the optimizer and categorical cross entropy was selected for the loss function.

### Transfer learning with pre-trained ConvNets

We evaluated four AI models that used transfer learning to implement state-of-the-art pre-trained convolutional neural networks. Transfer learning is a subfield of machine learning and artificial intelligence which uses the learned weights of an already trained model to solve a different problem instead of starting the training process of a model over from scratch, this approach saves time and computational costs^24^. Transfer learning for computer vision problems is normally executed by applying pre-trained ConvNet architectures (e.g. VGG, ResNet, Xception etc.) that were trained on large benchmark datasets (e.g. ImageNet^19^) to solve a particular problem. The pre-trained convolutional neural networks (ConvNets) allow us to build AI models for image classification with relatively high accuracy and diagnostic performance even if the target dataset is small or if the people tackling the problem do not have the required expertise to train a CNN from scratch. Pre-trained image classification networks are trained on a subset of the ImageNet database used in the ImageNet Large-Scale Visual Recognition Challenge (ILSVRC)^25^. Four pre-trained convolutional neural networks; ResNet152^15^, VGG19^16^, Xception^17^, and NASNETLarge^18^ were used in this study. ResNet152 is a newer version of ResNet (Residual Network), which is a convolutional neural network built and trained by Microsoft^15^. VGG19, which has a depth of 19 layers, was established by the University of Oxford in 2014^16^. Xception which was built and trained by Google, is a novel deep convolutional neural network inspired by Inception^17^. It slightly outperformed InceptionV3 (GoogLeNet)^26^ on the ImageNet database. Lastly, NASNETLarge is a state-of-art neural image classification model built and trained by Google in 2018^18^. The pre-trained models are freely accessible through the Keras Application (https://keras.io/api/applications/), which is a deep learning library. After the pre-trained models were chosen, we repurposed the knowledge that had already been learned; the layers, features, weights, and biases by fine-tuning to generate the correct outputs for our problem. Batch sizes of 20 and 10 epochs were determined as the optimum values for the pre-trained CNNs in consideration of time and computational costs. When compiling each model, Nadam was chosen as the optimizer and categorical cross entropy was selected for the loss function.

### Statistical analysis

To evaluate the classification performance of the AI models, area under the curve (AUC) of the receiver operating characteristic curve (ROC), precision, recall with accuracy, and f1-score were utilized.

True positive (TP): the number of cases where the class was correctly identified versus the rest of classes.

False positive (FP): the number of cases where the class was incorrectly identified versus the rest of classes.

True negative (TN): the number of cases correctly identified as healthy or other cancer type.

False negative (FN): the number of cases incorrectly identified as healthy or other cancer type.

$$Accuracy = \frac{TP + TN}{{TP + TN + FP + FN}}$$

$$precision = \frac{TP}{{TP + FP}}$$

$$recall = \frac{TN}{{TN + FP}}$$

$$F_{1} \;score = 2 \cdot \frac{precision \cdot recall}{{precision + recall}}$$

$$precision_{micro} = \frac{{\mathop \sum \nolimits_{k = 1}^{n} TP_{k} }}{{\mathop \sum \nolimits_{k = 1}^{n} TP_{k} + \mathop \sum \nolimits_{k = 1}^{n} FP_{k} }}, \quad n = 5$$

$$precision_{macro} = \frac{1}{n}\mathop \sum \limits_{k = 1}^{n} precision_{k} , \quad n = 5$$

All statistical analyses were performed using the scikit-learn library (https://scikit-learn.org/) from Python version 3.8.3 (https://www.python.org/).

## Data Availability

The dataset used in this study might be shared upon reasonable request to Jung Wook Yang, MD, PhD.
